# Improving blood product utilization at an ambulatory surgery center: a retrospective cohort study on 50 patients with lumbar disc replacement

**DOI:** 10.1186/s13037-019-0226-0

**Published:** 2019-12-19

**Authors:** Benjamin C. Dorenkamp, Madisen K. Janssen, Michael E. Janssen

**Affiliations:** 1 Orthopedic Surgery Residency, McLaren Greater Lansing, 401 W Greenlawn Ave, Lansing, MI 48910 USA; 2 College of Osteopathic Medicine, Rocky Vista University, 8401 S Chambers Rd, Parker, CO 80134 USA; 3 Center for Spine & Orthopedics, 9005 Grant St #200, Denver, CO 80229 USA

**Keywords:** Spine surgery, Ambulatory surgery center, Lumbar total disc arthroplasty, Anterior lumbar surgery, Blood product management

## Abstract

**Background:**

There is minimal literature discussing anterior lumbar spine surgery in ambulatory surgery centers (ASCs). The main concern with the anterior approach to the lumbar spine is the potential for injury to great vessels. In our facility, there are two units of crossmatched blood available in addition to cell saver during the procedure. We retrospectively looked at 50 cases of lumbar total disc arthroplasty (TDA) in our ASC to determine utilization of blood products.

**Methods:**

Medical records of 50 consecutive patients who underwent a lumbar TDA at a single ASC were reviewed. Surgeries completed at the ASC were all transferred from the post anesthesia care unit to an attached convalescence care center which allows up to 3 days of observation. Patients who had either a 1 or 2 level lumbar TDA were included in the study. Data consisting of demographics, American Society of Anesthesiologist Physical Status Classification System, length of stay, estimated blood loss, cell saver volume, transfusion, perioperative and postoperative complications were recorded. Preoperative, perioperative and postoperative medical records were reviewed.

**Results:**

Medical records of 50 consecutive patients were reviewed. The mean age was 40.86 ± 9.45. Of these, 48 (96%) had a 1-level lumbar TDA, 1(2%) had a 2-level lumbar TDA, 1 (2%) had a lumbar TDA at L4/5 and an anterior lumbar interbody fusion at L5/S1. There were no mortalities; no patient had recorded perioperative complications. No patients received allogeneic blood transfusion, 4 (8%) were re-transfused with cell saver (2 receiving approximately 400 ml and 2 receiving approximately 200 ml of re-transfused blood). All 50 (100%) were discharged home in stable condition. We had 30-day follow-up data on 35 of 50 patients. Of the 35 patients reviewed, three (8.5%) of the patients were readmitted to the hospital. One additional patient was seen in the emergency department and discharged home after negative testing. No patient was readmitted for post-operative anemia.

**Conclusion:**

The routine use of both cell saver and crossmatched blood in the operating suite for lumbar TDA may be an over-utilization of healthcare resources. In our review of 50 patients, we had no need for transfusion of allogeneic packed red blood cells (PRBCs) and only four of the 50 patients had enough blood output for re-transfusion from the cell saver. This opens the conversation for alternatives to crossmatched PRBCs being held in the operating room. Such alternatives may be the use of cell salvage, only type O blood in a cooler for each patient or keeping type O blood on constant hold in ASCs.

## Background

With increased pressure to improve the value of care in the United States, many surgeons are taking procedures to ambulatory surgery centers (ASCs). ASCs have demonstrated that they are more cost effective than traditional hospitals with little to no increase in morbidity and mortality [1–3]. However, there is minimal data regarding anterior lumbar surgeries via a retroperitoneal approach in this setting.

One of the main concerns with the anterior retroperitoneal approach to the lumbar spine is the concern for the safety of the great vessels. These are located in the retroperitoneal space and require mobilization and protection during the procedure. An exposure for a standard anterior lumbar interbody fusion (ALIF) requires a more limited dissection when compared to a lumbar total disc arthroplasty (TDA). This is because an ALIF can be placed from a more lateral position where as a TDA requires a larger window to access the midline of the intervertebral space for anatomic placement of the implant.

In most studies, incidence of major vascular injury during open anterior lumbar spinal surgery is reported at < 5% with some outliers reporting major venous injury up to 18% of cases [4–6]. Fantini et al. performed a retrospective chart review of 345 operations and reported that the left common iliac vein is the most commonly injured structure. They also reported that when a vascular injury occurred the mean estimated blood loss was 1510 ml +/− 854 mL with the lowest blood loss in their series being 250 mL and highest blood loss being 5000 ml. Of those patients with vessel injuries, they reported no mortalities [4].

The original maximum surgical blood order schedule (MSBOS) by Friedman et al. in 1975 estimated that a spinal fusion should have 4–5 units of blood on hold prior to going to surgery [7]. Modern blood ordering protocols look at the crossmatch to transfusion ratio (C:T) of 2:1 as a guideline to facilitate crossmatching fewer RBC units and thus more efficient management of blood bank inventories [8]. With utilization of cell saver technology as well as modern hemostatic agents, we feel that we are overutilizing our blood product resources and crossmatching an excessive number of RBCs causing our C:T ratio to exceed the recommended 2:1.

In this study, we retrospectively looked at blood product utilization in 50 patients undergoing lumbar total disc arthroplasty at a single ambulatory surgery center. Our study aims to assess the crossmatch to transfusion ratio and identify areas that can increase our efficiency regarding blood product utilization.

## Methods

This study was approved by Hospital Corporation of America -HealthONE Institutional Review Board, in Denver, Colorado. Study number 1312373–1. We performed a retrospective observational cohort study of 50 consecutive patients who underwent a lumbar TDA at a single ASC. Inclusion criteria included: 1 or 2 level lumbar total disc replacement between 2007 and 2018, surgery performed at a musculoskeletal surgery center and patients greater than 18 years old. Patients that were excluded included previous anterior lumbar surgery, lumbar disc replacement at an inpatient hospital and patients less than 18 years of age. All patients prior to surgery received a complete blood count and were typed and crossed with 2 units of PRBCs that were placed in a cooler at the surgery center (Table 1)**.** It is our routine practice to obtain a CT angiogram of the upper abdomen and pelvis prior to anterior lumbar surgery to define the anatomy of retroperitoneal structures for our access surgeon [9]. Results were only reviewed from patients that underwent surgery at the ASC. Thus, we are unable to determine if a certain subset of patients were excluded because of anatomic abnormalities seen on CT scan. Surgery was performed by an orthopedic spine surgeon and a general surgeon with significant experience in the anterior retroperitoneal approach to lumbar spine. All cases utilized intraoperative cell saver and were transfused with autologous blood if cell saver volume was over 200 ml. Fluoroscopy was used to verify implant placement. Surgeries were completed at the ASC and were all transferred from the post anesthesia care unit (PACU) to an attached convalescence care center which allows up to 3 days of observation. A postoperative hemoglobin was only performed if patient had subjective symptoms of anemia or labile vital signs. Data consisting of demographics, American Society of Anesthesiologists Physical Status classification (ASA), length of stay, estimated blood loss (EBL), cell saver volume, transfusion, perioperative and postoperative complications were recorded. Preoperative, perioperative and postoperative medical records were included.

**Table 1:** Pre-Op Hemoglobin

Hemoglobin g/dL	n	%
< 12	0	0.0%
12–13.9	9	18.0%
14–15.9	28	24.0%
16–17.9	12	24.0%
Unknown	1	2.0%

## Results

Medical records of 50 patients were reviewed. For all 50 patients we had complete preoperative, perioperative, as well as postoperative medical records. 30-day post-discharge data was available for 35 of the 50 patients included in the study. The mean age was 40.86 ± 9.45. Of these, 48 (96%) of patients had a 1-level lumbar TDA, 1(2%) patients had a 2-level lumbar TDA, 1 (2%) patient had a lumbar TDA at L4/5 and an anterior lumbar interbody fusion at L5/S1 (Table 2). There were no mortalities or perioperative complications. None of our patients received allogeneic blood transfusion, 4 (8%) were re-transfused from cell saver (2 patients receiving approximately 400 ml and 2 patients receiving approximately 200 ml of re-transfused blood) (Table 3). All 50 (100%) patients were discharged home in stable condition. Average blood loss was 119 mL with a maximum blood loss of 490 mL and was estimated based on cell saver collection (Table 4). Only one of the 50 patients had a postoperative hemoglobin laboratory value checked after one episode of dizziness with low oxygen saturation and it resulted at 10.9 g/dL; the patient was asymptomatic following inspiratory spirometry therapy and discharged home in stable condition. One patient was noted to be hypotensive the night of surgery, but responded to fluid resuscitation. The next morning, the patient was asymptomatic and discharged home on postoperative day one; it was determined the patient did not need a postoperative hemoglobin check. One patient became hypoxic postoperatively and responded to Narcan and a nasal airway, no further postoperative complications were reported during the patient’s stay. One patient presented 3 weeks postoperatively with lower extremity radicular pain and was taken back to surgery at the ASC for a microdiscectomy at the same level of the lumbar TDA but was not re-admitted to the hospital. One patient did not have record of cell saver volume, but he was asymptomatic postoperatively and was discharged home postoperative day one with no complications. Of the medical records reviewed, we had 30-day follow-up data on 35 of 50 patients. Of the 35 patients reviewed, three (8.5%) patients were readmitted to the hospital (1 pneumonia, 1 acute kidney injury, and 1 for pain and nausea). One additional patient was seen in the emergency department for testicular swelling with all testing reported negative and was discharged home. No patient was readmitted for postoperative anemia.

**Table 2:** Patient Demographics

		n	%
Sex	Male	30	60.0%
	Female	20	40.0%
Age (years)	20–29	7	14%
	30–39	15	30.0%
	40–49	17	34.0%
	50–60	11	22.0%
Current Smoker	Yes	15	30.0%
	No	35	70.0%
BMI	< 18.5	0	0.0%
	18.5–24.9	20	40.9%
	25–29.9	21	42.0%
	30–34.9	9	18.0%
	> 40	0	0.0%
ASA	1	14	28.0%
	2	33	66.0%
	3	3	6.0%
	4	0	0.0%
	5	0	0.0%
Surgical Levels	L4/L5	17	34.0%
	L5/S1	31	62.0%
	L4-S1	1	2.0%
	L4/L5 TDA & L5/S1 Fusion	1	2.0%
Length of Stay (LOS)	< 23	10	20
	24–36	29	58
	37–48	10	20
	48–72	0	0.0
	> 72	1	2.0

**Table 3:** Cell Saver Re-transfusion

Re-transfused (mL)	n	%
0	46	92.0%
200	2	4.0%
400–420	2	4.0%

**Table 4:** Estimated Blood Loss (EBL)

EBL (mL)	n	%
0–99	19	38
100–199	24	48
200–299	5	10
➢ 300	2	4

## Discussion

While there is an increased pressure to decrease cost within the medical field, we as a medical profession must ensure that we continue to provide the most efficient care while maintaining equivalent safety when compared to surgery in the hospital setting. The push to move increasingly complex surgeries to ASC has been met with skepticism by the public. This is demonstrated in a USA Today article that cited a string of complications at various surgery centers [10]. It is the responsibility of the medical community to demonstrate that the procedures we perform are being done safely with scientific data to support our protocols.

At our institution, we have been successfully performing lumbar total disc arthroplasty via an anterior retroperitoneal approach in our ASC since 2007. In our study we found that no patients required transfusion at any timepoint during their stay at the ASC or convalescent care center. We also observed out of the 50 cases performed we had no vascular injuries noted in the record. All patients were discharged home with no immediate postoperative complications.

It is our standard protocol for an access surgeon to perform the retroperitoneal approach. Phan et al. performed a systematic review looking at complication rates with anterior lumbar interbody fusions (ALIF) performed with and without an access surgeon. This study found that vascular injuries were higher in the group that utilized an access surgeon compared to those with no access surgeon. However, they also report that there were higher implant complications in the group that did not utilize access surgeons. The higher implant complication rate was likely secondary to inadequate exposure of the lumbar spine [11]. This becomes even more important when discussing lumbar total disc arthroplasty as it is imperative to have access to the midline of the vertebral body for correct placement of the implant. Mobbs et al. retrospectively looked at 227 patients undergoing an anterior retroperitoneal approach and advocated that having an access surgeon added safety and efficiency to anterior lumbar surgery [12]. While we understand that an access surgeon may not be available to all spine surgeons and does add significant cost to the procedure, we feel that the skillset of an access surgeon allows for the safest and most reproducible surgical outcomes.

To aid in a safe anterior surgical approach, a preoperative CT angiogram of the pelvis is obtained to identify any anatomic anomalies. In a prospective cohort by Datta et al., the surgical plan was modified in 21% of their study patients due to anatomic variations found on CT scan [9]. It is our routine practice for patients having their surgery performed as an inpatient or at the ambulatory surgery center to have a preoperative CT scan prior to anterior lumbar surgery. This is particularly important in the ambulatory setting to ensure a safe and reproducible surgery in a setting where limited resources are available.

The original maximum surgical blood ordering schedule (MSBOS) was developed to increase the effective shelf life of a unit of PRBCs and decrease workload on blood bank personnel. In order to preserve the effective shelf life of a unit of blood, we must decrease the time a unit is in an assigned (crossmatched) status [7]. In order to implement the goal of the MSBOS, it is suggested that institutions maintain a crossmatch to transfusion ratio (C:T) of 2:1 [8, 13]. In the original MSBOS, it was estimated that a spinal fusion should have 4–5 units of PRBCs crossmatched but did not delineate the type or location of the spinal fusion. Since 1975, many new surgeries and techniques for spinal fusion have been developed and the MSBOS must be carefully reviewed to allow it to be utilized to its full potential [7].

A study in 2013 by Frank et all at John’s Hopkins University revised the MSBOS and proposed that retroperitoneal surgery should have 2 units of PRBCs typed and crossmatched, whereas thoracic, lumbar and sacral spinal fusions should have 4 units of PRBCs typed and crossmatched [13]. In the case of anterior TDA, we utilize a retroperitoneal approach with much less bony work compared to posterior spinal fusions-- allowing this procedure to fall more favorably into a retroperitoneal category. Our current practice follows Frank and colleague’s recommendation to crossmatch 2 units of PRBCs per patient that undergoes an anterior lumbar TDA. However, our data shows that we have had zero transfusions after 50 surgeries making our C:T ratio much higher than 2:1, suggesting over-utilization of crossmatching resources.

The most modest reduction in blood transfusions, as managed through the patient blood management processes (PBM), has shown a tremendous reduction in costs. Cell salvage as well as hemostatic agents has been found to be very efficacious in lowering costs associated with allogeneic transfusions [14]. It is important to point out that spinal fusion is one of the most common surgeries requiring blood transfusion, yet overall the allogeneic blood transfusion rate is found to be only 6.5%, as reported in Yoshiihara and Yonekoka’s retrospective study from 2014 [14].

Because of the risk of potential severe intraoperative blood loss and vascular injury during spinal surgery, cell saver technology has been commonly used and has lowered the need for allogeneic transfusions. The intraoperative autologous blood transfusion system has been a critical component to the success of ASC facilities, enhancing the safety of the procedures and effectively avoiding the need for blood transfusions, while remaining cost effective [15]. This is not only a cost-effective technology, but helps mitigate the adverse effects and bad patient outcomes from allogeneic transfusions during surgery. For spinal surgery, blood transfusions are considered a major outcome determinant [16]. Multicenter, prospective process cost analysis in the US and Europe showed that the cost to administer a RBC transfusion is several fold the cost of production of RBC products [17]. The use of the cell saver technology has drastically improved the ability of ASCs to remain cost effective, while maintaining high standards of safety.

In addition to the cell saver technology, the use of advanced topical hemostatic agents and tranexamic acid (TXA) therapies can also decrease blood loss, reduce need for blood transfusions, decrease operation time, and thus reduce the cost of surgery. These absorbable hemostatic matrix products provide a cost-effective option to bleeding during spinal surgery, in attempts to avoid transfusions. Price et al. demonstrated that these products can decrease the cost of stay and overall care for patients undergoing spinal surgery [18]. In addition, David et al., reported that the cost impact of the flowable hemostatic agents was substantial, even when taking into consideration that multiple units of the product are often required in spinal surgeries. A systematic review by Willner et al. discussed the effectiveness of the TXA therapy as having a 49% reduction in blood loss and required fewer blood transfusions all without significant side effects. The TXA therapies have been found to effectively reduce intraoperative blood loss without the risks of venous thromboembolism (VTE) [16].

In preparation for a lumbar TDA, each patient at our institution receives a type and cross with two units of PRBCs placed on hold and transferred to the surgery center the day of surgery in a cooler. Given that none of our 50 patients required transfusion, we argue that it would be safe to have our patients typed and screened, reserving the crossmatching for those patients that have a clinically significant antibody as identified in the type and crossmatch. The electronic crossmatch eliminates the step of testing donor RBC with patient plasma and decreases the time required to crossmatch a unit of RBC to < 1 min [19]. Current lifespan of RBCs is 42 days at 1–6 degrees Celsius. Therefore, it is crucial to limit the time products are taken out of circulation [20]. Given the routine use cell saver technology and access to hemostatic agents, we could decrease the amount of time crossmatched units spend out of general circulation and decrease the workload on blood bank staff. To maintain safety in an emergent scenario of a major vessel injury, we would have 2 units of uncrossmatched type-O red cells (UORBC) at the ASC. Safety of UORBC has been reported in Journal of Trauma by Dutton et al. who looked at 581 units of UORBC in 161 patients and showed no acute hemolytic transfusion reactions, and this is because the O blood is known to be safe in a patient with a previously negative antibody screen [21]. This would allow equivalent safety precautions with less resource utilization.

Limitations to this study include the following. With only 50 patients in our series, we understand that this study may be underpowered and subject to type II error. We had complete data for all 50 patients during their perioperative period until discharge. However, we only had complete 30-day data for 35 of 50 patients. While we do accept that having incomplete 30-day medical records is a limitation to the study our primary objective was in regard to perioperative complications in relation to acute blood loss.

We understand that surgery is not a perfect science and there will always be risk of vascular injury in anterior lumbar surgery. Further limitations of this study include the lack of discussion of patients’ preoperative hemoglobin and taking into account the inflating effect of high altitude on hemoglobin. Further, we do not discuss the science and methodology behind the cell saver technology and that the technology is limited by dilution with roughly only 50–55% of the total solution being the hematocrit; when a patient receives 250 ml of blood back from the cell saver, roughly 137 ml of blood is returned.

This study retrospectively looked at patients that had been previously screened and cleared to have surgery at an ambulatory surgery center. This puts our data at risk for bias. This is particularly true regarding the use of a preoperative CT scan because we are unable to determine if any patients were disqualified for surgery in the ambulatory setting based on their anatomic variations identified by the access surgeon prior to surgery. Finally, this system works well because our study includes more-experienced surgeons and because the ASC was reasonably close to a hospital that could provide the blood products.

## Conclusion

We conclude that performing anterior lumbar surgery via a retroperitoneal approach at an ASC is not only safe but could likely be done with less resource utilization of blood products. With modern cell saver technology and hemostatic agents, we are able to perform surgeries with less blood loss and thus utilizing our resources more efficiently. By only stocking UORBC at the ASC, we would decrease time that crossmatched blood spent out of general circulation and decrease the workload on blood bank staff. We propose that this data be used as preliminary information demonstrating a need for larger controlled studies to evaluate the safety and utilization of resources in anterior lumbar spine surgery in the ambulatory setting.

## Data Availability

The datasets used and/or analyzed during the current study are available from the corresponding author on reasonable request.

## Abbreviations

ALIF: Anterior Lumbar Interbody Fusion
ASA: American Society of Anesthesiologists Physical Status Classification System
ASC: Ambulatory Surgery Center
C:T: Crossmatch to Transfusion Ratio
CT: Computerized Tomography
EBL: Estimated Blood Loss
MSBOS: Maximum Surgical Blood Order Schedule
PACU: Post Anesthesia Care Unit
PBM: Patient Blood Management Processes
PRBC: Packed Red Blood Cells
RBC: Red Blood Cells
TDA: Total Disc Arthroplasty
TXA: Tranexamic Acid
UORBC: Uncrossmatched Type-O Red Cells, VTE: Venous Thromboembolism
